# The peripheral monocyte count is associated with the density of tumor-associated macrophages in the tumor microenvironment of colorectal cancer: a retrospective study

**DOI:** 10.1186/s12885-017-3395-1

**Published:** 2017-06-05

**Authors:** Masatsune Shibutani, Kiyoshi Maeda, Hisashi Nagahara, Tatsunari Fukuoka, Shigetomi Nakao, Shinji Matsutani, Kosei Hirakawa, Masaichi Ohira

**Affiliations:** Department of Surgical Oncology, Osaka City University Graduate School of Medicine, 1-4-3 Asahi-machi, Abeno-ku, Osaka City, Osaka Prefecture 545-8585 Japan

**Keywords:** Colorectal cancer, Monocyte, Tumor-associated macrophage

## Abstract

**Background:**

Inflammation is widely recognized to play an important role in cancer progression, and the peripheral monocyte count has been reported to correlate with the prognosis in patients with colorectal cancer. This is based on the hypothesis that the peripheral monocyte level and the density of tumor-associated macrophages (TAMs) in the cancer microenvironment correlate with each other. However, the influence of TAMs on the prognosis and the correlation between the peripheral monocyte count and the density of TAMs have not yet been elucidated.

**Methods:**

A total of 168 patients with stage II/III colorectal cancer were enrolled in this study. Preoperative blood samples were obtained at the time of the diagnosis before surgery. The expression of TAMs in the cancer microenvironment was assessed by immunohistochemistry.

**Results:**

The progression-free and overall survival rate were significantly worse in the high-TAMs group than in the low-TAMs group (*p* = 0.0012 and *p* = 0.0207, respectively). The peripheral monocyte count was significantly associated with the number of TAMs (correlation coefficients: 0.202, *p* = 0.047).

**Conclusions:**

The peripheral monocyte count was associated with the density of the TAMs, which created a microenvironment favorable for cancer development and were correlated with a poor prognosis. Therefore, the peripheral monocyte count is a useful prognostic marker reflecting the status of the tumor microenvironment.

## Background

Inflammation is widely recognized to play an important role in cancer progression [1], and various inflammatory markers have been reported as useful prognostic markers in patients with various types of cancer [2–6]. The peripheral monocyte count, which is one such inflammatory marker, has been reported to correlate with the prognosis in patients with prostate, breast and colorectal cancer [7–9]. Moreover, in our previous study, the same results were obtained in colorectal cancer [10].

Tumor-associated macrophages (TAMs) are macrophages that exist within the tumor microenvironment and are derived from circulating monocytes [11, 12]. There are two kinds of macrophage phenotypes: the M1 phenotype has antitumor activity, whereas the M2 phenotype promotes cancer progression [13, 14]. Most TAMs have an M2-like phenotype and promote metastasis, angiogenesis, and immunosuppression [15].

The concept of the peripheral monocyte count being a useful prognostic marker in cancer patients is based on the hypothesis that the peripheral monocyte count reflects the density of TAMs in the cancer microenvironment [9, 10, 16]. However, the influence of TAMs on the prognosis and the correlation between the peripheral monocyte count and the density of TAMs have not been elucidated.

In this study, we evaluated the prognostic significance of TAMs and clarified the correlation between the peripheral monocyte count and the density of TAMs in patients with colorectal cancer.

## Methods

### Patients

A total of 168 patients with stage II/III colorectal cancer were enrolled in this study. All patients underwent potentially curative surgery for colorectal cancer at the Department of Surgical Oncology of Osaka City University between 2007 and 2009. Patients who received preoperative therapy, underwent emergency surgery for perforation/obstruction, or who had inflammatory bowel disease were excluded from this study.

The patient characteristics are listed in Table 1. A total of 85 males and 83 females were included in this study. The median age of the patients at the initial surgery was 67 years old (range: 26 to 90 years old). Ninety patients had primary tumors located in the colon, and 78 had primary tumors located in the rectum. The resected specimens were pathologically classified according to the seventh edition of the UICC TNM classification of malignant tumors [17]. The distribution of cancer stages was as follows: stage II, 92 patients; stage III, 76 patients. All patients were followed up regularly with physical and blood examinations, including measurements of the levels of tumor markers, such as carcinoembryonic antigen (CEA) and carbohydrate antigen 19–9 (CA19–9), and mandatory screening using colonoscopy and computed tomography until December 2016 or death.

**Table 1:** Patient characteristics

Gender	
Male	85
Female	83
Age (years)	
Median (range)	67 (26–90)
Location of primary tumor	
Colon	90
Rectum	78
Tumor depth^a^	
T1–3	109
T4	59
Tumor diameter (cm)	
Median (range)	5.0 (1.0–11.0)
Histological type	
Well, Moderately	154
Poorly, Mucinous	14
Lymphatic involvement	
Negative	40
Positive	128
Venous involvement	
Negative	137
Positive	31
Lymph node metastases	
Negative	92
Positive	76
Peripheral monocyte count (/mm3)	
Median (range)	348 (28–719)
The number of TAMs (/field)	
Median (range)	7.67 (0.67–58.67)

*TAMs* tumor-associated macrophages

^a^:According to the UICC. TNM Classification of Malignant Tumors (Seventh edition)

### Blood sample analysis

Preoperative blood samples were obtained at the time of the diagnosis before surgery. The differential white blood cell count was analyzed using an XE-5000 hematology analyzer (Sysmex, Kobe, Japan) in accordance with the manufacturer’s protocol.

### Immunohistochemistry

CD163 has been used as a specific marker to identify M2 macrophages [13, 14]. Surgically resected specimens were retrieved to perform immunohistochemistry. Sections 4 μm in thickness were deparaffined and rehydrated. The sections were then subjected to endogenous peroxidase blocking in 1% H_2_O_2_ solution in methanol for 15 min. Antigen retrieval was performed by autoclaving the sections at 105 °C for 10 min in Dako Target Retrieval Solution (Dako, Glostrup, Denmark). Serum blocking was performed with 10% normal rabbit serum for 10 min. After H_2_O_2_ and serum blocking, the slides were incubated with primary mouse monoclonal anti-CD163 antibody (1:200 dilution; Leica Biosystems, Newcastle Upon Tyne, UK) at room temperature for 1 h. The secondary antibody was biotin-labeled rabbit anti-mouse IgG (1:500; Nichirei, Tokyo, Japan). Detection was performed with a DAB kit (Histofine simple stain kit; Nichirei). The sections were counterstained with hematoxylin.

### Immunohistochemical evaluations

**Fig. 1:**
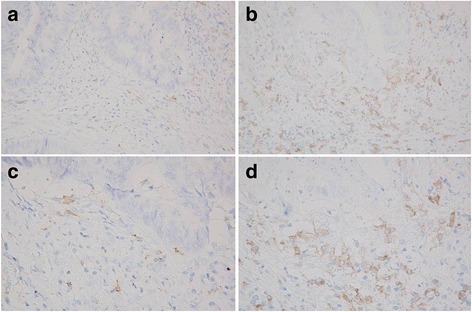
The immunohistochemical expression of CD163, an M2 macrophage-specific marker. **a** A low density of TAMs (100×) **b** A high density of TAMs (100×) **c** A low density of TAMs (400×) **d** A high density of TAMs (400×)

Immunohistochemical evaluations were carried out by two independent pathologists blinded to the clinical information. The number of immunoreactive macrophages at the invasive margin was counted with a light microscope in a randomly selected field at a magnification of 400× (Fig. 1). The mean of the values obtained in five different areas was used for the data analysis. According to the median TAM value, we set 8.0 as the cut-off value for the evaluation of TAMs and classified patients into a high-TAMs group and a low-TAMs group.

### Statistical analyses

The significance of the correlations between TAMs and the clinicopathological characteristics were analyzed using the *χ* ^*2*^ test and Fisher’s exact test. The duration of the survival was calculated using the Kaplan-Meier method. Differences in the survival curves were assessed using the log-rank test. A multivariate analysis was performed using the Cox proportional hazard model. Associations between peripheral monocyte count and the density of TAMs in the tumor microenvironment were evaluated by Spearman’s rank correlation coefficient. All of the statistical analyses were conducted using the SPSS software package for Windows (SPSS Japan, Tokyo, Japan). *P* values of <0.05 were considered to indicate statistical significance.

### Ethical considerations

This research conformed to the provisions of the Declaration of Helsinki. All patients were informed of the investigational nature of this study and provided their written informed consent. This retrospective study was approved by the ethics committee of Osaka City University (approved No.926).

## Results

### Correlations between the density of TAMs and the clinicopathological factors

The density of TAMs showed no significant relationship with any of the clinicopathological parameters, except for the histological type (Table 2).

**Table 2:** Correlations between the density of TAMs and clinicopathological factors

	TAM		
	Low	High	*p*-value
Age (years)			
<70	50	59	
≥70	35	24	0.108
Gender			
Male	37	47	
Female	48	35	0.089
Tumor depth^a^			
T1–3	58	51	
T4	27	32	0.420
Histological type			
Well, moderate	82	72	
Poorly, mucinous	3	11	0.027
Tumor diameter			
<5 cm	58	49	
≥5 cm	27	34	0.262
Lymphatic involvement			
Negative	20	20	
Positive	65	63	1.000
Venous involvement			
Negative	70	67	
Positive	15	16	0.844
Lymph node metastasis			
Negative	46	46	
Positive	39	37	0.878
CEA			
≤5 ng/ml	56	49	
>5 ng/ml	29	34	0.426
CA19–9			
≤37 U/ml	76	71	
>37 U/ml	6	12	0.211

*TAMs* tumor-associated macrophages, *CEA* carcinoembryonic antigen, *CA19–9* carbohydrate antigen 19–9

^a^:According to the UICC. TNM Classification of Malignant Tumors (Seventh edition)

### Survival analyses according to the density of TAMs

**Fig. 2:**
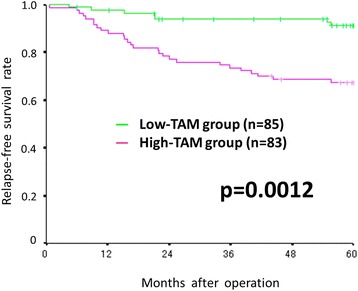
Kaplan-Meier survival curves for the relapse-free survival according to the density of TAMs. The relapse-free survival rate was significantly worse in the high-TAMs group than in the low-TAMs group (*p* = 0.0012)

**Fig. 3:**
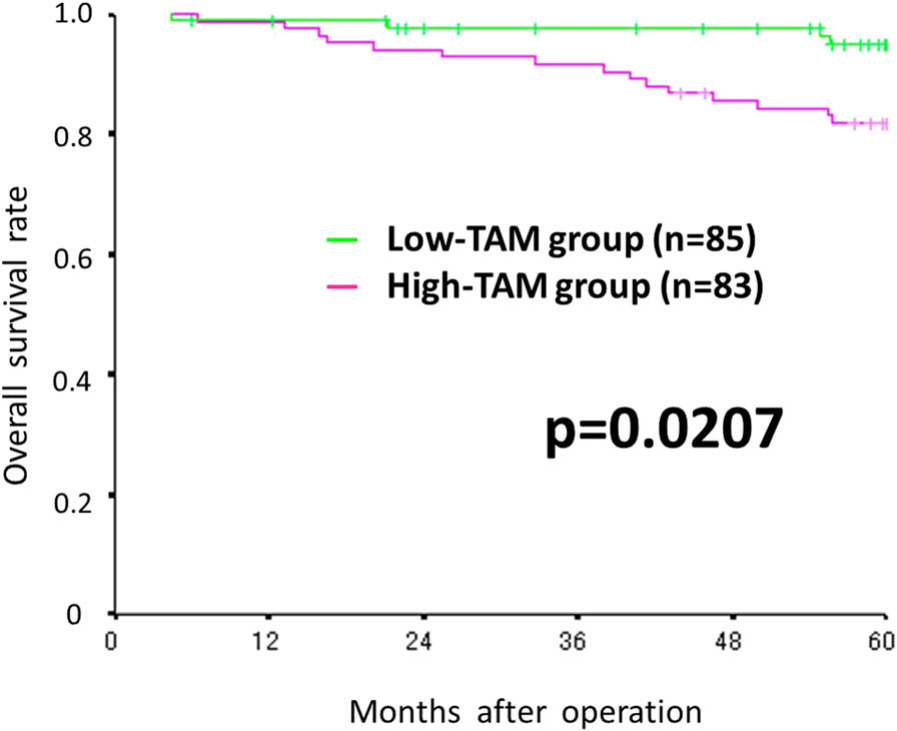
Kaplan-Meier survival curves for the overall survival according to the density of TAMs. The overall survival rate was significantly worse in the high-TAMs group than in the low-TAMsgroup (*p* = 0.0207)

The progression-free survival rate was significantly worse in the high-TAMs group than in the low-TAMs group (*p* = 0.0012) (Fig. 2). The overall survival rate was also significantly worse in the high-TAMs group than in the low-TAMs group (*p* = 0.0207) (Fig. 3).

### Prognostic factors influencing the survival

The correlations between the progression-free survival and the clinicopathological factors are shown in Table 3. According to the results of a univariate analysis, the progression-free survival showed significant relationships with the density of TAMs (*p* = 0.002), lymphatic involvement (*p* = 0.011), lymph node metastasis (*p* = 0.001), CEA (*p* = 0.014), and CA19–9 (*p* < 0.001). A multivariate analysis indicated that the density of TAMs (hazard ratio: 3.692; 95% confidence interval: 1.743–7.822; *p* = 0.001) and lymph node metastasis (hazard ratio: 2.251; 95% confidence interval: 1.131–4.481; *p* = 0.021) were independent prognostic factors for the progression-free survival.

**Table 3:** Correlations between the relapse-free survival and clinicopathological factors

	Univariate analysis			Multivariate analysis		
	HR	95% CI	*P*-value	HR	95% CI	*P*-value
Age (≥70 years vs. <70 years)	1.611	0.882–2.942	0.121			
Gender (Male vs. Female)	1.657	0.894–3.071	0.109			
Tumor depth (T4 vs. T1–3)	1.617	0.879–2.976	0.123			
Histological type (Poorly, Mucinous vs. Well, Moderately)	2.092	0.879–4.978	0.095			
Lymphatic involvement (Positive vs. Negative)	3.837	1.367–10.767	0.011	2.563	0.879–7.471	0.085
Venous involvement (Positive vs. Negative)	1.857	0.953–3.621	0.069			
Lymph node metastasis (Positive vs. Negative)	3.016	1.589–5.724	0.001	2.251	1.131–4.481	0.021
CEA (>5 ng/ml vs. ≤5 ng/ml)	2.123	1.165–3.870	0.014	1.332	0.667–2.660	0.417
CA19–9 (>37 U/ml vs. ≤37 U/ml)	3.764	1.821–7.777	<0.001	1.821	0.782–4.242	0.165
TAM (High vs. Low)	2.973	1.493–5.920	0.002	3.692	1.743–7.822	0.001

*HR* hazard ratio, *CI* confidence interval, *TAMs* tumor-associated macrophages, *CEA* carcinoembryonic antigen, *CA19–9* carbohydrate antigen 19–9

The correlations between the overall survival and the clinicopathological factors are shown in Table 4. According to the results of a univariate analysis, the overall survival showed significant relationships with the density of TAMs (*p* = 0.027), age (*p* = 0.036), venous involvement (*p* = 0.010), lymph node metastasis (*p* = 0.024), CEA (*p* = 0.021), and CA19–9 (*p* = 0.017). A multivariate analysis indicated that the density of TAMs (hazard ratio: 4.123; 95% confidence interval: 1.464–11.610; *p* = 0.007), age (hazard ratio: 3.355; 95% confidence interval: 1.373–8.200; *p* = 0.008), and venous involvement (hazard ratio: 3.911; 95% confidence interval: 1.540–9.936; *p* = 0.004) were independent prognostic factors for the overall survival.

**Table 4:** Correlations between the overall survival and clinicopathological factors

	Univariate analysis			Multivariate analysis		
	HR	95% CI	*P*-value	HR	95% CI	*P*-value
Age (≥70 years vs. <70 years)	2.366	1.058–5.289	0.036	3.355	1.373–8.200	0.008
Gender (Male vs. Female)	1.250	0.565–2.765	0.582			
Tumor depth (T4 vs. T1–3)	1.387	0.616–3.124	0.430			
Histological type (Poorly, Mucinous vs. Well, Moderately)	1.660	0.495–5.573	0.412			
Lymphatic involvement (Positive vs. Negative)	2.461	0.733–8.258	0.145			
Venous involvement (Positive vs. Negative)	2.987	1.306–6.831	0.010	3.911	1.540–9.936	0.004
Lymph node metastasis (Positive vs. Negative)	2.641	1.135–6.148	0.024	1.729	0.699–4.273	0.236
CEA (>5 ng/ml vs. ≤5 ng/ml)	2.563	1.149–5.717	0.021	1.258	0.513–3.085	0.616
CA19–9 (>37 U/ml vs. ≤37 U/ml)	3.117	1.226–7.928	0.017	2.127	0.720–6.281	0.172
TAM (High vs. Low)	2.841	1.128–7.152	0.027	4.123	1.464–11.610	0.007

*HR* hazard ratio, *CI* confidence interval, *TAMs* tumor-associated macrophages, *CEA* carcinoembryonic antigen, *CA19–9* carbohydrate antigen 19–9

### Correlation between the peripheral monocyte count and the number of TAMs in the tumor microenvironment

**Fig. 4:**
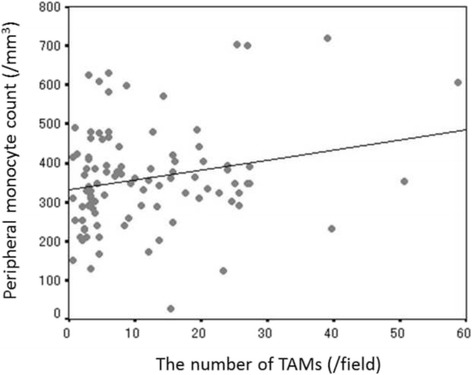
The correlation between the peripheral monocyte count and the number of TAMs in the tumor microenvironment (correlation coefficient: 0.202, *p* = 0.047)

The peripheral monocyte count was significantly associated with the number of TAMs (correlation coefficient: 0.202, *p* = 0.047) (Fig. 4).

## Discussion

We found that a high density of TAMs in the cancer microenvironment was associated with a poor prognosis in patients with colorectal cancer. We also found that the peripheral monocyte count was associated with the density of TAMs in the cancer microenvironment. These results may explain the reason why the peripheral monocyte count functions as a prognostic marker in patients with colorectal cancer.

Increasing evidence suggests that stromal cells, such as tumor-infiltrating lymphocytes, TAMs, and cancer-associated fibroblasts, in the cancer microenvironment are associated with cancer progression. TAMs were first reported in the early 1980s [18] and have been extensively studied, with their process of differentiation and function now clear. Monocytes differentiate into macrophages after recruitment from the peripheral blood to the tumor [11, 12, 19]. They can be divided into two main phenotypes: M1 type and M2 type. These polarizations are adjusted by cytokines, such as macrophage-colony-stimulating factor (M-CSF), transforming growth factor (TGF)-β, interleukin (IL)-6 and IL-10 in the cancer microenvironment [20, 21]. M1 macrophages have antitumor activity, whereas M2 macrophages play an important role in invasion, metastasis, angiogenesis, and immunosuppression, which lead to cancer progression [15, 22]. M2-macrophages play an important role in tumor progression and metastasis via angiogenesis through their production of angiogenic factors such as vascular endothelial growth factor (VEGF) [23, 24], and play a role in tumor invasion via a matrix metalloproteinase (MMP)-dependent mechanism through their production of tumor necrosis factor-alpha (TNF-α) [25]. Moreover, M2 macrophages are responsible for immunosuppression through their inhibition of the T cell function via the programmed cell death-1 (PD-1)/programmed cell death-ligand 1 (PDL1) pathway and their production of immunosuppressive cytokines such as interleukin (IL)-10 [26]. Because the majority of TAMs have an M2-like phenotype [27], the high density of TAMs in the cancer microenvironment is associated with a poor prognosis.

In previous reports, the peripheral monocyte count and the lymphocyte-to-monocyte ratio have been reported to be useful prognostic markers [9, 10, 16, 28, 29]. This was based on the hypothesis that the peripheral monocyte count was associated with the density of TAMs in the cancer microenvironment. However, few reports have described the correlation between the peripheral monocyte count and the density of TAMs in the cancer microenvironment. In the present study, the peripheral monocyte count was shown to correlate with the density of TAMs in the cancer microenvironment, suggesting that inflammatory markers such as the peripheral monocyte count might be surrogate markers reflecting the status of the cancer microenvironment. A peripheral blood cell count is a quick, easy, and inexpensive assay to perform and is often carried out as a routine examination. We hope that peripheral inflammatory markers will be applied clinically as biomarkers in patients with colorectal cancer in the future.

The median peripheral monocyte count, which was obtained 5 years after operation from patients who had been relapse free, was 321 (range: 118–504). This value was significantly lower than the preoperative peripheral monocyte count (*p* < 0.001, paired *t*-test). The mechanism underlying the increase in the peripheral monocyte count of cancer patients is considered to be as follows. Chemokines (such as CCL2), which are produced by cancer cells, promote the recruitment of peripheral monocytes to the cancer microenvironment, thereby promoting the recruitment of monocytes from the bone marrow to peripheral blood.

Several limitations associated with the present study warrant mention. First, we evaluated a relatively small number of patients, and the study design was retrospective. Second, factors other than the response of the host to the cancer, which affect the systemic inflammation, were not assessed. Third, M1 macrophages, which are also derived from circulating monocytes, were not considered in this study, although most macrophages in the cancer microenvironment are reported to be M2 macrophages, and the impact of M1 macrophages on the cancer microenvironment is likely negligible. Fourth, we did not verify the polarization of the macrophages in this study. Further studies are needed to elucidate the mechanisms underlying M1/M2 polarization in the cancer microenvironment. By co-culturing the peripheral monocytes and cancer cell lines, we can confirm that most monocytes polarized to the M2 phenotype in the cancer microenvironment and investigate the types of cytokines that are involved in polarization.

## Conclusions

In conclusion, our results showed that the peripheral monocyte count was associated with the density of the TAMs, which created a microenvironment favorable for cancer development and were correlated with a poor prognosis, in the cancer microenvironment. Therefore, the peripheral monocyte count is considered to be a useful prognostic marker reflecting the status of the tumor microenvironment.

## Abbreviations

CA19–9: Carbohydrate antigen 19–9
CEA: Carcinoembryonic antigen
CI: Confidence interval
HR: Hazard ratio
IL: Interleukin
M-CSF: Macrophage-colony-stimulating factor
TAMs: Tumor-associated macrophages
TGF-β: Transforming growth factor-β
